# The association between social media use and well-being during quarantine period: testing a moderated mediation model

**DOI:** 10.3389/fpsyg.2023.1265496

**Published:** 2023-11-03

**Authors:** Leling Zhu, Shuaijie Xiao, Xinyu Yan, Shuijia Zhou, Jiemin Yang, Jiajin Yuan

**Affiliations:** ^1^Institute of Brain and Psychological Science, Sichuan Normal University, Chengdu, China; ^2^Faculty of Psychology and Education, Université Libre de Bruxelles, Brussels, Belgium; ^3^Sichuan Key Laboratory of Psychology and Behavior of Discipline Inspection and Supervision, Sichuan Normal University, Chengdu, China

**Keywords:** social media use, humor style, subject well-being, optimism, COVID-19

## Abstract

**Objectives:**

Social media use (SMU) increased dramatically during COVID-19 due to policies such as long-term quarantine. Given that SMU has complex effects on individuals’ well-being, this study aimed to explore the relationship between SMU and subjective well-being and the influencing factors in the context of the pandemic in China.

**Methods:**

A total of 895 adults (413 males) in different risk areas across China participated in this study. They provided self-reported data on subjective well-being, social media use, adaptive humor, and other demographic variables.

**Results:**

It revealed that SMU was positively associated with individual well-being, an effect partially mediated by the score of adaptive humor. Furthermore, the effect of SMU on adaptive humor was moderated by trait optimism, with the effect more robust in high (vs. low) optimistic individuals.

**Conclusion:**

This study explored the positive effects of SMU on individuals’ well-being, suggesting that individuals may better cope with negative experiences and maintain well-being under quarantine by showing more adaptive humor on social media.

## 1. Introduction

To stop the spread of the COVID-19 virus efficiently, China adopted the dynamic zero-COVID policy, but many studies indicated that strict measures like lockdowns and long-time quarantines are always accompanied by negative experiences including fear, anxiety, distress, and a decrease in well-being (Reizer et al., 2021; Diotaiuti et al., 2023). This raises an urgent question about what people could do to better cope with these negative experiences and maintain their subjective well-being during the long-term quarantines.

Given the fact that offline communication was limited during the quarantine period, online social media became the major channels for people to acquire relative information, basic supplies, and a sense of belonging (Sitar-Taut et al., 2021). Prior studies suggest that social media use has a complex impact on individuals’ well-being, and whether the impact is positive or negative depends on many factors, including the pattern of social media use (Erfani and Abedin, 2018; Hanley et al., 2019; Diotaiuti et al., 2022). Due to the quarantine policy, people have to use social media much more actively and frequently than usual to compensate for the lack of offline activities, which means that social media use may positively impact individuals’ well-being in this unique context.

After looking through the content during this period, researchers noticed that pandemic-related jokes and memes circulated rapidly on social media (Bischetti et al., 2021). Such humor, consisting of visual collages composed of phrases and images taken from popular media, served as a frequent and highly valued element of online communication (Kuipers, 2002; Laineste and Voolaid, 2017). Undoubtedly, the creation and circulation of these humor materials are deeply influenced by the use of social media. Moreover, researchers suggest that humor is useful for people to cope with adverse circumstances and life stressors (Kuipers, 2002; Sebba-Elran, 2021). Therefore, we infer that the use of social media might facilitate the creation and circulation of humor, and further help people to cope with life stressors.

However, according to the work of Martin et al. (2003), the use of humor could be divided into four types: (1) self-enhancing type refers to the relatively benign use of humor to enhance the self; (2) affiliative humor means to enhance one’s relationships with others by relatively benign humor; (3) aggressive type emphasizes the use of humor to enhance the self at the expense of others; and (4) self-defeating humor refers to the use of humor to enhance relationships at the expense of the self. Only the first two types of humor use can enhance well-being and mental health (adaptive use of humor), and the rest harm well-being and mental health (maladaptive use of humor), which is supported by empirical studies (Reizer et al., 2022). Therefore, we hypothesize that adaptive humor may mediate the effects of social media use on well-being, and more specifically that adaptive humor plays a positive role.

Furthermore, personality factors such as trait optimism can play a key role in the creation and circulation of humor, in that optimistic individuals tend to interpret situations more humorously and are more likely to create and circulate humor materials compared to pessimistic people (Torres-Marín et al., 2022). Optimistic trait reflects an individual’s overall positive expectations for the future, which has been confirmed to have a positive effect on subjects’ well-being, even when people are under quarantine (Forgeard and Seligman, 2012; Reizer et al., 2022). According to the conservation of resources theory, optimism is one of the most important personal resources, and individuals with relatively few resources are more likely to be aggressive and irrational when faced with resource loss and have difficulty gaining resources (Hobfoll et al., 2018). Evidence also proves that the adaptive use of humor is positively correlated with optimism (Marrero et al., 2020). In other words, when facing stressful events such as the pandemic quarantine, individuals with high optimism traits are more likely to create and appreciate humorous materials because they are equipped with more individual resources. Therefore, we further hypothesize that individuals with high optimism traits are more likely to generate adaptive humor use on social media.

**Figure 1:**
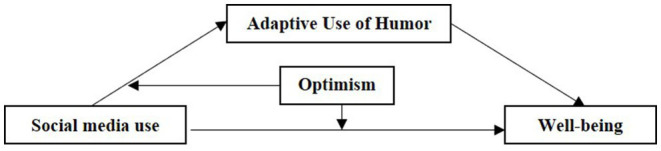
Conceptual framework.

Hence, this study aimed to explore the relationship between social media use and well-being in the context of epidemic quarantine, and further investigate the roles of humor and trait optimism in this pathway. Based on the literature reviewed above, we would examine a moderated mediation model as illustrated in Figure 1. We hypothesize that: (1) social media use would be positively related to the adaptive use of humor; (2) adaptive use of humor would mediate the association between social media use and well-being; (3) optimism would moderate the association between social media use and adaptive use humor; and the association between social media use and well-being.

## 2. Methods

### 2.1. Participants

We collected data through an online questionnaire during the pandemic. Our sample consisted of 895 participants (479 females, 413 males and two did not specify their genders). 872 subjects’ ages ranged from 18 to 40 years, 20 subjects were older than 40 and 3 were under 18 years. 78% of subjects were in the low-risk area,^1^ 15% in the medium-risk area,^2^ and 7% in the high-risk area.^3^ 53% of subjects were under long-time quarantine at the time of the survey.

Participants were informed about the aim of the study and were asked for their consent to participate. The study received ethical approval from the host university to launch the study.

### 2.2. Measures

#### 2.2.1. Social media use

Social media use is measured by the Chinese version of the Social Media Use Intensity Questionnaire developed by Ellison et al. (2007) (Sun et al., 2016). The first two items measure the number of social media friends and the average time spent on social media per day (multiple choice), while the remaining six items measure the strength of the emotional connection to social media and the extent to which social media is integrated into the individual’s life, presenting on a five-point Likert scale, ranging from 1 (strongly disagree) to 5 (strongly agree). The scores on these questions were converted into standardized scores first due to differing item scale ranges and then averaged. In this study, the Cronbach α is 0.827 for eight items.

#### 2.2.2. Subjective well-being and general well-being

Subjective well-being was calculated by summing standardized scores for life satisfaction and positive affect and then subtracting a standardized score for negative emotions. To improve the completion rate of the questionnaire and reduce the response burden of the participants, we adopt the five-item Satisfaction With Life Scale (Diener et al., 1985), items are presented on a seven-point Likert-type scale (Cronbach α = 0.92). An 18-item Chinese revision of the Positive and Negative Scale was used to measure affect, nine items assessed positive affect (Cronbach α = 0.95), the rest assessed negative affect (Cronbach α = 0.92), both are presented on a five-point Likert-type scale (Qiu et al., 2008).

General well-being was measured with the simplified version of the General Well-Being Schedule (Cronbach α = 0.86), which is the first 18 questions of Duan Jianhua’s revised version (Fazio, 1977; Duan, 1996).

#### 2.2.3. Adaptive use of humor

The subscale of affiliate humor and self-enhancing humor of the Chinese adaption of Humor Styles Questionnaire (HSQ; Martin et al., 2003; Wang, 2011) is presented on a five-point Likert-type scale, ranging from 1 (totally agree) to 5 (totally disagree). Fifteen items assessed adaptive humor––affiliative humor style (α = 0.84) and self-enhancing humor (α = 0.83). Adaptive use of humor was calculated by summing scores for affiliate humor and self-enhancing humor.

#### 2.2.4. Optimism

Optimism was measured using the 10-item Life Orientation Test-Revised (LOT-R; Scheier et al., 1994), and four items out of 10 were not scored because they were interfering items. Items are presented on a five-point Likert-type scale, ranging from 1 (strongly disagree) to 5 (strongly agree). The optimism score comprises the sum of the responses (α = 0.75).

### 2.3. Confirmatory factor analysis

Confirmatory factor analysis (CFA) was conducted in Mplus 8.3 (Muthén and Muthén, 2017) to test the measurement models for the present study. The measurement model for adaptive humor comprising affiliate humor and self-enhancing humor exhibited good model fit after we deleted one item with standardized loading lower than 0.45 [χ^2^(76) = 431.337, CFI = 0.906, RMSEA = 0.072, and SRMR = 0.053]. Likewise, the results revealed a good model fit for subjective well-being [χ^2^(227) = 1096.465, CFI = 0.936, RMSEA = 0.065, and SRMR = 0.061]. In sum, the results revealed a good model fit for all scales, indicating that all scales had sound measurement properties.

### 2.4. Data analysis

First, descriptive statistics and bivariate pairwise correlations were performed for the variables of social media use, well-being, humor types, and optimism. Then, separate regression analyses were carried out with the two types of well-being as the outcome variables. Second, a statistical mediation analysis was conducted in SPSS PROCESS 3.4 to examine the indirect effects of social media use on well-being via humor types (Model 4). Third, based on the results of the mediation analysis, a moderated mediation analysis (Model 7 and Model 8) was conducted to investigate the moderating role of optimism in the pathway between social media use and humor types.

## 3. Results

### 3.1. Common method bias

The data collected in this study was controlled for common method bias through the anonymous collection, and reverse item scoring. We employed Harman’s single-factor test to check common method bias (Podsakoff et al., 2003) and the result showed that the first factor only explained 23.24% of the variance, below 40%, which means that common method bias did not appear to pose a threat in interpreting our results.

### 3.2. Descriptive and correlation

The descriptive statistics of the study variables and their bivariate correlations are presented in Table 1. As expected, social media use was positively correlated with adaptive psychological outcomes (subjective well-being and general well-being), adaptive use of humor, and optimism. Adaptive use of humor was positively correlated with optimism and adaptive psychological outcomes.

**Table 1:** Means, standard deviations, and zero-order bivariate correlations.

M ± SD	1	2	3	4	5	6	7	8	9	10	
PA	3.18 ± 1.06	1.00	−0.383^**^	0.654^**^	0.733^**^	0.669^**^	0.393^**^	0.513^**^	0.483^**^	0.278^**^	0.411^**^
NA	1.93 ± 0.83	−0.383^**^	1.00	−0.405^**^	−0.501^**^	−0.632^**^	−0.347^**^	−0.298^**^	−0.356^**^	−0.117^**^	−0.390^**^
SWLS	22.94 ± 7	0.654^**^	−0.405^**^	1.00	0.989^**^	0.675^**^	0.411^**^	0.506^**^	0.491^**^	0.256^**^	0.423^**^
SWB	24.2 ± 8.12	0.733^**^	−0.501^**^	0.989^**^	1.00	0.734^**^	0.441^**^	0.534^**^	0.523^**^	0.269^**^	0.458^**^
GWB	83.53 ± 16.31	0.669^**^	−0.632^**^	0.675^**^	0.734^**^	1.00	0.536^**^	0.530^**^	0.582^**^	0.288^**^	0.613^**^
HA	29.77 ± 5.55	0.393^**^	−0.347^**^	0.411^**^	0.441^**^	0.536^**^	1.00	0.672^**^	0.942^**^	0.397^**^	0.524^**^
HS	22.98 ± 3.93	0.513^**^	−0.298^**^	0.506^**^	0.534^**^	0.530^**^	0.672^**^	1.00	0.881^**^	0.406^**^	0.503^**^
AHSQ	52.75 ± 8.7	0.483^**^	−0.356^**^	0.491^**^	0.523^**^	0.582^**^	0.942^**^	0.881^**^	1.00	0.437^**^	0.562^**^
SMU	-	0.278^**^	−0.117^**^	0.256^**^	0.269^**^	0.288^**^	0.397^**^	0.406^**^	0.437^**^	1.00	0.346^**^
OP	22.22 ± 3.61	0.411^**^	−0.390^**^	0.423^**^	0.458^**^	0.613^**^	0.524^**^	0.503^**^	0.562^**^	0.346^**^	1.00

PA, Positive affect; NA, Negative affect; SWLS, Life satisfaction; SWB, Subjective well-being; GWB, General well-being; HA, Affiliate humor; HS, Self-enhancing humor; AHSQ, Adaptive humor; SMU, Social media use; OP, Trait optimism. *p* < 0.05^**^p < 0.01.

### 3.3. Regression analysis

Multiple regression was used to examine the overall effect of social media use on GWB and SWB respectively, controlling for gender, age risk level, and epidemic prevention measures and the results showed that social media use had a significant positive predictive effect on both types of well-being [SWB: *R*^2^ = 0.121, *F*_(5,889)_ = 24.435, B = 0. 265, *t*_(894)_ = 8.429, *p* = 0. 000; GWB: *R*^2^ = 0. 121, *F*_(5,889)_ = 24.392, B = 0. 283, *t*_(894)_ = 9.002, *p* = 0. 000]. These results are in line with our hypothesis. However, when participants were separated by risk level and gender, social media use tended to negatively predict the general well-being of female participants in high-risk areas (GWB: B = −0.013, *p* = 0.949).

### 3.4. Indirect associations between social media use and well-being

The results of the mediation analysis are shown in Table 2. All variables in the model are brought into the regression equation by *Z*-value, and controls for age, gender, risk level, and epidemic prevention measures. In line with our hypotheses, the results of the statistical mediation analysis showed a statistically significant indirect effect of social media use on well-being through adaptive humor (GWB: B = 0.242, SE = 0.023, 95% CI = [0.200, 0.289]; SWB: B = 0.211, SE = 0.021, 95% CI = [0.172, 0.254]).

**Table 2:** Results of the mediation analysis for adaptive humor: indirect and direct effect (Model 4).

Independent variable	Mediating variable	Dependent variable	Std. Est.	95% CI
*The indirect and direct effects*				
SMU→	AHSQ	→GWB	**0.437**	[0.378,0.496]
	AHSQ		**0.554**	[0.495,0.613]
*Indirect effect*				
SMU→	AHSQ	→GWB	**0.242**	[0.200,0.289]
*Direct effect controlling for the indirect effect*				
SMU→		→GWB	0.041	[−0.017,0.100]
*The indirect and direct effects*				
SMU→	AHSQ		**0.437**	[0.378,0.496]
	AHSQ	→SWB	**0.482**	[0.420,0.543]
*Indirect effect*				
SMU→	AHSQ	→SWB	**0.211**	[0.172,0.254]
*Direct effect controlling for the indirect effect*				
SMU→		→SWB	0.055	[−0.007,0.116]

Std Est., Standardized estimate; 95% CI, 95% bias-corrected bootstrap confidence interval; SMU, Social media use; AHSQ, Adaptive humor; GWB, General well-being; SWB, Subjective well-being statistically significant results at *a* = 0.05 are in boldface; → indicates the direction of the pathway between two variables. R2AHSQ = 0.205; R2GWB = 0.364; R2SWB = 0.305.

### 3.5. Moderating role of optimism

**Figure 2:**
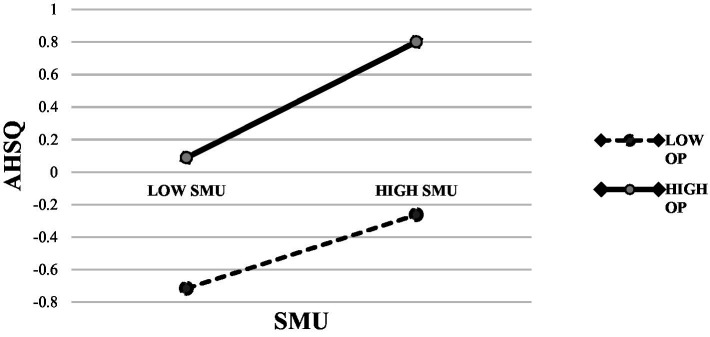
Moderating effect of optimism trait (OP).

Based on the mediation model examined above, we further tested whether the optimism trait would moderate the association between social media use and well-being. The results of the moderated mediation analysis for social media use using Model 8 only showed a statistically significant interaction between SMU and OP in predicting AHSQ (Index of moderated mediation: GWB: 95% CI [0.001, 0.044], Std. Est. = 0.021; SWB:95% CI [0.001, 0.047], Std. Est. = 0.023). Therefore, we re-conducted moderated mediation analysis using Model 7, and the results are shown in Table 3 (For brevity, regression coefficients for control variables are not presented in the table). The results of the simple slope test indicated that the association between social media use and adaptive use of humor was stronger among participants with high optimism trait (B = 0.357, SE = 0.043, *p* = 0.000) than those with low optimism (B = 0.226, SE = 0.033, *p* = 0.000; Figure 2).

**Table 3:** Results of the mediation analysis for adaptive humor: indirect and direct effect (Model 7).

Independent variable	Mediating variable	Dependent variable	Std. Est.	95% CI
*The indirect and direct effects*				
SMU→	AHSQ		**0.292**	[0.235,0.348]
OP→	AHSQ		**0.467**	[0.412,0.522]
SMU^*^OP→	AHSQ		**0.065**	[0.014,0.116]
	AHSQ	→GWB	**0.554**	[0.259,0.381]
*Indirect effect gave the average level of optimism(M = 0)*				
SMU→	AHSQ	→GWB	**0.161**	[0.122,0.204]
*Direct effect controlling for the indirect effect gave the average level of optimism*				
SMU→		→GWB	0.041	[−0.017,0.100]
*The indirect and direct effects*				
SMU→	AHSQ		**0.292**	[0.235,0.348]
OP→	AHSQ		**0.467**	[0.412,0.522]
SMU^*^OP→	AHSQ		**0.065**	[0.014,0.116]
	AHSQ	→SWB	**0.482**	[0.420,0.543]
*Indirect effect gave the average level of optimism (M = 0)*				
SMU→	AHSQ	→SWB	**0.140**	[0.106,0.178]
*Direct effect controlling for the indirect effect gave the average level of optimism*				
SMU→		→SWB	0.055	[−0.007,0.116]

Std. Est., Standardized estimate; 95% CI, 95% bias-corrected bootstrap confidence interval; SMU, Social media use; OP, Trait optimism; AHSQ, Adaptive humor; GWB, General well-being; SWB, Subjective well-being; statistically significant results at *a* = 0.05 are in boldface; → indicates the direction of the pathway between two variables. R2AHSQ = 0.404; R2GWB = 0.364; R2SWB = 0.305.

## 4. Discussion

Prior studies have examined the complex impact of social media use on mental health (Erfani and Abedin, 2018; Hanley et al., 2019; Diotaiuti et al., 2022). Given the fact that social media use compensated for the lack of offline activities when people faced strict prevention measures, social media use may play a buffer role during this particular period. To examine this assumption, we launched an online questionnaire to assess social media use, humor, optimism traits, and individual well-being in different risk areas in China during COVID-19. It is confirmed that social media use contributes to individuals’ mental health, which is mediated by adaptive humor, and optimism traits moderate the effect of social media use on adaptive humor.

### 4.1. The association between SMU and well-being

The results showed that social media use is a significant positive predictor of individuals’ well-being in the specific context, which is consistent with previous research (Sitar-Taut et al., 2021). This finding may be due to the fact that social media compensated for the lack of offline interpersonal interactions, and satisfied individuals’ social needs during quarantine, thus having a positive impact on well-being. Surprisingly, we found a negative tendency between social media use and the general well-being of female participants in high-risk areas, although this tendency is not statistically significant.

According to the relevant policy, residents in high-risk areas were required to stay at home until the risk level had decreased, which took at least 14 days, whereas residents of low- and medium-risk level areas were allowed to engage in offline interpersonal interactions under certain conditions. Researchers suggested that there were gender differences in the sources of well-being, with only women’s well-being being predicted by involvement in balanced and mutually satisfying relationships (Reid, 2004). Apparently, the relevant policy constrained women in high-risk areas to attain relationship sources offline. However, when social media was the only source of well-being for women, the content on social media may not always be supportive of young women, who make up 70% of the female participants in high-risk areas. For example, it is been confirmed that there is a significant relationship between young women’s frequency of social media use and body dissatisfaction, drive for thinness, and low self-esteem during quarantine, which negatively predict well-being (Vall-Roqué et al., 2021). In other words, both sources of well-being are at risk for the younger group of women. In addition, residents in high-risk level areas can only order daily necessities online, and they have had to constantly monitor information in online chat groups to make sure they order and receive them on time. That is, social media use is associated with more work rather than entertainment. The situation might be harder for women who have already started a family and have children, as they usually shoulder more domestic and parental work (Sayer, 2005; Schmidt et al., 2021), so it is not hard to understand the negative relationship between social media use and well-being of female participants in high-risk areas.

In summary, for women in high-risk areas, in addition to having relatively fewer resources for well-being, their use of social media is associated with heavy psychological stressors, and these stressors may negatively impact their well-being. Considering that women are more susceptible to negative emotions in life situations (Yuan et al., 2009), we infer that it is the combination of relatively fewer resources, heavy stressors, and females’ unique traits leading to the negative tendency between social media use and well-being. Therefore, it is necessary to consider the unique characteristics of women and pay more attention to their mental health when major social events occur in the future.

### 4.2. The mediating and moderating effects

Our results found that adaptive humor was positively related to social media use and well-being, and mediated the effects of social media use on well-being. The humor related to critical social events, defined as disaster humor, always emerges right after its outbreak and circulates fast on social media. Prior research suggested that disaster humor is not only a reflection of social media culture but also a collective narrative shaped by netizens (Kuipers, 2002). That is, the more individuals use social media, the more likely they engage in creating and circulating humor materials. Furthermore, humor is always considered to be an effective coping strategy, which elicits positive emotions that counteract negative ones, reduces stress, and increases compassion, connection, and empathy (Horn et al., 2019; McBride and Ball, 2022). Adaptive humor is confirmed to enhance well-being and mental health empirically (Reizer et al., 2022). Therefore, adaptive humor can help promote the positive effects of social media use on well-being.

The results of the moderated mediation model analysis partly confirmed our hypothesis that the intensity of social media use promotes well-being through adaptive humor and that optimism moderates the effect of social media use on adaptive humor, meaning that this effect is more pronounced in groups with high optimism. That is, individuals with high optimism traits process external information from a positive perspective, which facilitates them to understand and appreciate humorous content on social media, and further engage in positive humor in daily life. This finding is also consistent with a previous study, which revealed that positive expectation about the future makes optimists take more adaptive strategies to solve problems (Hamilton et al., 2015). To sum it up, optimism facilitates the display of adaptive humor on social media, further improving well-being during the unique period when people heavily rely on social media.

It should be noted that the moderation effect is relatively small. Considering that optimism is only one kind of personal resource, optimism may play a relatively smaller role when facing immense stressors like COVID-19. The overall resources of individuals are limited and if stressors persist over time, individual resources will finally be exhausted and enter a state of stress, as illustrated by the study on emotional burnout during the Wuhan city closure (Shao et al., 2021). Therefore, in addition to paying attention to personal resources, we should also value other social supports, such as interpersonal relationships and comprehensive psychological intervention resources provided by the whole society, so that we can ensure that the majority of the population has relatively adequate social support to cope with the crisis.

### 4.3. Implications and limitations

The present study has potential theoretical and practical implications. On the one hand, it offers new insights into the correlation between social media use and well-being. In the present study, we investigated the predictive effect of social media use on mental health and further explored the mechanisms from the perspective of adaptive humor during quarantine. These findings enrich the existing research on the promotion effect of social media use on psychological health and enhance our understanding of the positive effects of social media use. On the other hand, these findings provide empirical evidence of how individuals can maintain mental health under the circumstances of critical public events like COVID-19. It is worth spending time on creating and circulating humor materials, which help narrate the situation adaptively. However, we still need to point out that although adaptive humor can help the general public cope better with stressful events, it may detract from the perceived seriousness and gravity of the epidemic itself and thus have other negative effects on individuals. As pointed out in a recent study, emotional regulation of negative emotions during the epidemic, while beneficial for maintaining mental health, may cause individuals to reduce necessary protective measures and threaten their physical health (Smith et al., 2021). How to balance mental health and physical health might be a point that could be of interest to future researchers. Besides, cultivating the trait of optimism is worth advocating, which can help individuals better appreciate and understand humorous materials. Previous literature has identified several methods to help people become more optimistic, including cognitive therapy, and the Penn Resiliency Program (Forgeard and Seligman, 2012).

We must acknowledge several limitations in this research. Firstly, a cross-sectional design was adopted and this may only be suggestive of causal inference. Secondly, although common method bias was not concerning in this study, self-report data is still inadequate. Therefore, readers should interpret the findings with caution, and it would be ideal for future research to manipulate the key variables and observe the changes. Thirdly, optimism is treated as a relatively stable personality in our study, while some other researchers regarded optimism as an explanatory style based on the attribution theory, which is equipped with different measure tools (Seligman, 1998). Finally, we focus on humorous behavior in a general sense, and future research should pay attention to investigating specific behaviors like posting and retweeting humorous content on social media.

## 5. Conclusion

Social media use increased dramatically during COVID-19 and was positively associated with individual well-being, an effect partially mediated by the score of adaptive humor. Furthermore, the effect of social media use on adaptive humor was moderated by trait optimism, with the effect more robust in high (vs. low) optimistic individuals. This study explores the positive effects of social media use on individuals’ well-being in the context of the pandemic and its potential mechanisms, providing a new idea for individuals to better cope with negative experiences and maintain their well-being by showing more adaptive humor on social media and cultivating the trait of optimism.

## Data availability statement

The raw data supporting the conclusions of this article will be made available by the authors, without undue reservation.

## Ethics statement

The studies involving humans were approved by the institutional research committee at Sichuan Normal University. The studies were conducted in accordance with the local legislation and institutional requirements. Written informed consent for participation in this study was provided by the participants’ legal guardians/next of kin. Written informed consent was obtained from the individual(s), and minor(s)’ legal guardian/next of kin, for the publication of any potentially identifiable images or data included in this article.

## Author contributions

LZ: Data curation, Formal Analysis, Investigation, Software, Visualization, Writing – original draft. SX: Data curation, Investigation, Visualization, Writing – review & editing. XY: Supervision, Writing – review & editing. SZ: Investigation, Writing – original draft. JYa: Conceptualization, Funding acquisition, Project administration, Resources, Supervision, Writing – review & editing. JYu: Funding acquisition, Resources, Writing – review & editing.

## Footnotes

^1^Low-risk areas refer to areas other than medium and high-risk areas in the counties (cities, districts, and banners) where medium and high-risk areas are located.

^2^Medium risk level areas are generally areas such as work places where cases and asymptomatic infected people stay for a certain time, and may have a risk of epidemic transmission.

^3^High-risk areas are generally areas where cases and asymptomatic infected persons live, and have a high risk of transmission of the outbreak.
