# Supplementary material for: The association between social media use and well-being during quarantine period: testing a moderated mediation model
Source: Front Psychol. 2023 Nov 3;14:1265496. doi: 10.3389/fpsyg.2023.1265496 (PMC10656604; doi:10.3389/fpsyg.2023.1265496)
Supplement: Supplementary file 1 [file Table_1.docx]

| **Results of the Mediation Analysis for Adaptive humor: Indirect and Direct Effect. (Model 8)** | | | | |
| --- | --- | --- | --- | --- |
| Independent Variable | Mediating Variable | Dependent Variable | Std.Est. | 95%CI |
| *The indirect and direct effects* | | | | |
| SMU→ | AHSQ |  | **0.292** | [0.235 ,0.348] |
| OP→ | AHSQ |  | **0.467** | [0.412 ,0.522] |
| SMU*OP→ | AHSQ |  | **0.065** | [0.014,0.116] |
|  | AHSQ | →GWB | **0.32** | [0.259 ,0.381] |
| OP→ |  | →GWB | **0.442** | [0.284 ,0.500] |
| SMU*OP→ |  | →GWB | 0.04 | [-0.007 ,0.087] |
| *Indirect effect gave the average level of optimsm(M=0)* | | | | |
| SMU→ | AHSQ | →GWB | **0.095** | [0.067 ,0.129] |
| *Direct effect controlling for the indirect effect gave the average level of optimsm* | | | | |
| SMU→ |  | →GWB | 0.02 | [-0.034 ,0.075] |
| *The indirect and direct effects* | | | | |
| SMU→ | AHSQ |  | **0.292** | [0.235 ,0.348] |
| OP→ | AHSQ |  | **0.467** | [0.412 ,0.522] |
| SMU*OP→ | AHSQ |  | **0.065** | [0.014,0.116] |
|  | AHSQ | →SWB | **0.351** | [0.282 ,0.420] |
| OP→ |  | →SWB | **0.256** | [0.191 ,0.322] |
| SMU*OP→ |  | →SWB | -0.017 | [-0.070 ,0.037] |
| *Indirect effect gave the average level of optimsm(M=0)* | | | | |
| SMU→ | AHSQ | →SWB | **0.102** | [0.073 ,0.137] |
| *Direct effect controlling for the indirect effect gave the average level of optimsm* | | | | |
| SMU→ |  | →SWB | 0.019 | [-0.043 ,0.081] |
| *Note*：Std Est. = standardized estimate, 95% CI = 95% bias-corrected bootstrap confidence interval;  SMU=social media use; OP= trait optimism; AHSQ=adaptive humor; GWB= general well-being; SWB= subjective well-being; statistically significant results at a = 0.05 are in boldface; → indicates the direction of the pathway between two variables. R^2^AHSQ =0.404; R^2^GWB=0.495; R^2^SWB=0.349 | | | | |

**Supplementary Table 1**
